# Influence of Weight Loss on Cognitive Functions: A Pilot Study of a Multidisciplinary Intervention Program for Obesity Treatment

**DOI:** 10.3390/brainsci12040509

**Published:** 2022-04-17

**Authors:** Emma Chávez-Manzanera, Maura Ramírez-Flores, Michelle Duran, Mariana Torres, Mariana Ramírez, Martha Kaufer-Horwitz, Sylvana Stephano, Lizette Quiroz-Casian, Carlos Cantú-Brito, Erwin Chiquete

**Affiliations:** 1 Obesity and Eating Disorders Clinic, Department of Endocrinology and Metabolism, Instituto Nacional de Ciencias Médicas y Nutrición Salvador Zubirán, Mexico City 14080, Mexico; emma.chavezm@incmnsz.mx (E.C.-M.); drastephano@gmail.com (S.S.); lizettequiroz@gmail.com (L.Q.-C.); 2 Laboratory of Neuropsychology and Cognition, School of Psychology, Universidad Nacional Autónoma de México, Mexico City 04510, Mexico; mau_ramz@comunidad.unam.mx (M.R.-F.); duran.gomez04@hotmail.com (M.D.); marianatbc456@gmail.com (M.T.); mrs95.7@hotmail.com (M.R.); 3 Department of Neurology and Psychiatry, Instituto Nacional de Ciencias Médicas y Nutrición Salvador Zubirán, Mexico City 14080, Mexico; carloscantu_brito@hotmail.com

**Keywords:** cognition, executive functions, obesity, weight loss, multidisciplinary program

## Abstract

There is a relationship between obesity and cognitive functioning. Our aim was to assess weight loss influence on global cognition and executive functioning (EF) in adults with obesity under a multidisciplinary weight loss program. In this six-month longitudinal study, we assessed 81 adults (age < 50 years) with body mass index (BMI) ≥ 30. EF and global cognitive performance were evaluated with the Montreal Cognitive Assessment (MoCA), Neuropsychological Battery of Executive Functions (BANFE-2) and Trail Making Test-Part B (TMT-B). Median age was 40.0 years (IQR: 31.5–47, 61% women), and the median BMI was 41.4 (IQR: 36.7–45.9). At a six-month follow-up, the mean weight loss was 2.67% (29.6% of patients achieved ≥5% weight loss). There was an improvement in EF evaluated with BANFE (*p* = 0.0024) and global cognition with MoCA (*p =* 0.0024). Women experienced more remarkable change, especially in EF. Weight loss did not correlate with cognitive performance, except for TMT-B (r-0.258, *p =* 0.026). In the regression analysis, only years of education predicted the MoCA score. This study showed that patients improved cognitive performance during the follow-up; nevertheless, the magnitude of weight loss did not correlate with cognitive improvement. Future studies are warranted to demonstrate if patients achieving ≥5% weight loss can improve cognition, secondary to weight loss.

## 1. Introduction

Obesity is widely recognized as a risk factor for the development of chronic degenerative diseases, such as metabolic, cardiovascular, cerebrovascular [1], mechanical [2], and mental [3]. It is also known that obesity conveys a high risk for mild cognitive impairment through dementia in older adults [4,5], middle-aged adults [6,7,8], and, even, young adults [9]. However, results from studies might be paradoxical, mainly in older adults [10,11]. These heterogeneous results emerge from methodological issues, participants’ age, ethnicity, metabolic comorbidities (e.g., fasting blood glucose, glucose variability, insulin resistance, hypertension, and other metabolic biomarkers) [12,13,14,15], pharmacotherapy (e.g., metformin) [16], and duration of long-term follow-up [9].

The cognitive impact in individuals with overweight or obesity is mostly observed in executive functioning (EF), attention, and memory [17,18,19]. EF is a superior cognitive process that regulates goal-oriented behavior, including inhibitory control, cognitive flexibility, working memory, decision-making, planning, and self-monitoring [19,20]. Alterations in EF influence eating behavior and the maintenance of obesity, resulting in a vicious cycle [21,22]. Therefore, early identification of EF disturbance is a primary objective to carry out interventions aimed at modifying the course of the disease [22]. Mitchell et al. estimated, in a metanalysis, a 21.9% progression rate of mild cognitive impairment to all-type dementia in the general population [23]. Nonetheless, numerous studies have demonstrated that weight loss improves not only cardiometabolic parameters [24,25,26], quality of life [27], and mortality reduction [1], but also cognitive functioning [28,29]. These findings may suggest that the risk of dementia might be modified with the intervention of multidisciplinary weight loss programs.

To our knowledge, there are no studies on the Mexican population that evaluate the impact of weight loss on global cognition and EF in young and middle-aged adults with obesity. Given the high prevalence of obesity in the young Mexican population, and the extended longevity that is expected in the near future, it is crucial to determine this association [30]. Therefore, this study aimed to assess global cognition and EF in young and middle-aged adults with obesity before and after a multidisciplinary weight loss program and to determine the correlation between cognitive performance and weight loss in patients who completed the program.

## 2. Materials and Methods

### 2.1. Population and Study Design

This is an observational study on patients that attended an Obesity Care Multidisciplinary Program (PAPO in Spanish) with a 6-months follow-up, at the Obesity Clinic at the Instituto Nacional de Ciencias Médicas y Nutrición Salvador Zubirán, which is a tertiary referral hospital in Mexico City, between October 2017 through March 2020.

The multidisciplinary program is carried out by physicians, nutritionists, psychologists, and psychiatrists. The PAPO structure consists of five individual sessions and two group courses in six months. The diet prescription was based on the Look AHEAD program, with a calorie goal of 1200–1800 kcal/day, depending on initial body weight. The exercise prescription consisted of a progressive plan, starting with 50 min a week (5 sessions a week lasting 10 min), until patients achieved a goal of 175 min of moderate to high intensity per week [31]. The evaluation and control of medical comorbidities were based on obesity guidelines [32]. We aimed at personally tailored psychological and psychiatric interventions to address each patient’s specific needs, including coaching, coping strategies, pharmacological treatment, and psychotherapy, if any psychopathology was found.

A total of 498 patients were evaluated for eligibility: men and women with obesity (BMI ≥ 30), aged 18 to 55 years, with ≥6 years of formal school education. Patients with use of psychotropic drugs (topiramate, benzodiazepines, tricyclic antidepressants, and monoamine oxidase inhibitors, as well as serotonin, norepinephrine, or dopamine reuptake inhibitors), pregnancy, renal or liver disease, uncontrolled thyroid disease, adrenal dysfunction, history of excessive alcohol use (women with three drinks/day, men with four drinks/day), heavy cigarette smoking (>15 cigarettes/day), neurologic injury or disease (e.g., neonatal hypoxia, cranial trauma, neuroinfection, brain tumor, epilepsy, stroke, cognitive disability, attention deficit hyperactivity disorder, and other conditions), or uncontrolled psychopathology (e.g., psychosis, obsessive-compulsive disorder, borderline personality, or major depressive disorder with suicidal thoughts) were excluded.

### 2.2. Demographic and Clinical Variables

We collected sociodemographic, anthropometric, medical, and biochemical information at the beginning of the program and at a 6-month follow-up. Demographic and medical history included sex at birth (i.e., women and men), age (years), schooling (years), cigarette smoking (categorized as absent, mild ≤ 5 cigarettes/day, moderate 6–15 cigarettes/day), and alcohol use (categorized as absent; mild: women with one drink/day and men with two drinks/day; moderate: women with two drinks/day and men with three drinks/day). Physical activity was obtained through the short version of the International Physical Activity Questionnaire [IPAQ], and patients were classified in three categories (mild < 600 Mets/week, moderate 600–1499 Mets/week, and high intensity ≥ 1500 Mets/week).

Medical comorbidities such as hypertension, diabetes, dyslipidemias, and obstructive sleep apnea (OSA) were collected through a detailed medical history and biochemical analyses, as well as OSA through the STOP-Bang Questionnaire. A semi-structured clinical interview for the DSM-5th edition was conducted to rule out psychopathology (anxiety disorder, major depressive disorder, and binge eating disorder).

Symptoms of anxiety and depression were assessed with a Hospital Anxiety and Depression (HAD) questionnaire: a standard score of <8 for anxiety and <7 for depression [33].

Anthropometric variables were measured with Medical Body Composition Analyzer (seca mBCA 514): determined weight (kg), fat mass (kg, %), and fat-free mass (kg, %). Waist circumference (cm) was measured with the Lufkin W606PM tape, while systolic and diastolic blood pressure were obtained with a mercury sphygmomanometer (mm/Hg).

The main biochemical analytes were glucose (mg/dL), total cholesterol (mg/dL), c-LDL (mg/dL), c-HDL (mg/dL), triglycerides (mg/dL), creatinine (mg/dL), ALT (mg/dL), AST (mg/dL), and TSH (mIU/L), which were analyzed with a Synchron Multi-Level Control Beckman Coulter kit.

### 2.3. Cognitive Assessments

We used the Montreal Cognitive Assessment (MoCA, Spanish version 12 November 2004; from 0 to 30 points, a scoring ≥26 considered normal performance) to assess global cognitive functioning. The reliability, correlation coefficient, sensitivity, and specificity tests in the Mexican population were 0.89, 0.955, 80%, and 75% respectively [34].

To assess EF, we used the Neuropsychological Executive Functions and Frontal Lobes Battery (in Spanish, Batería Neuropsicológica de Funciones Ejecutivas y Lóbulos Frontales, BANFE), which comprises a set of tests that are widely used by the international community due to their high convergent and clinical validity to measure EF that are supported by clinical neuropsychology and neuroimaging studies, showing a high correlation with cognitive processes and brain activity. The BANFE is divided into two subtests: BANFE-2 for EF and frontal lobes assessment and the BANFE-neuropsychological questionnaire of self-perception (BANFE-NQS) to assess behavioral changes typically related to frontal damage. BANFE-2 classified EF according to the main neuroanatomical and functional correlate within the prefrontal cortex (PFC) regions: anterior PFC, medial orbital PFC, and dorsolateral PFC (from 0 to 146 points, depending on the anatomical region studied). The tests included in BANFE measure the following EF subdomains: planning, decision making, verbal and visuospatial working memory, cognitive flexibility, inhibitory control, and metacognition (Supplementary Tables S1 and S2). All raw scores were converted to normalized values through a table endorsed by the Center for Assistance, Teaching and Psychoneurocognitive Research Aidyne (standardized sample of 450 Mexicans adjusted by age and years of education). The battery performance was dichotomized into normal performance (≥85 points) and low performance (<85 points), and was stratified into four levels of severity: severe (≤69 points), mild to moderate (70–84 points), normal (85–115 points), and superior (≥116 points) [35]. BANFE-NQS (from 0 to 40 points, with a higher score indicating worse status) was used to assess self-perception in the following fields: self-consciousness (<3 points), interests and motivations (<3 points), behavioral control (<4 points), frustration tolerance (<3 points), mood (<3 points), executive functioning (<4 points), and the total score (<15 points).

Additionally, Trail Making Test-Part B was used to measure processing speed and mental flexibility; performance in this test was assessed by completion time. A shorter time indicates a faster information processing speed, and a range from 75 to 273 s is considered normal. This test involves connecting an alternating sequence of numbered and lettered circles. All neuropsychologic tests were performed by three trained standardized neuropsychologists (M.D., M.T., and M.R.), further supervised by a fourth expert neuropsychologist (M.R.F), who was blind to the clinical information of the patients.

### 2.4. Statistical Analysis

Sample size was determined with the aim of finding a Pearson’s correlation of at least 30% between weight loss and improvement in EF, with an alpha of 0.05 and a power of 0.80. Categorical data are presented as relative frequencies in the form of proportions, while continuous data are presented as medians with their respective interquartile ranges (IQR), depending on the distribution. The Kolmogorov–Smirnov test assessed the equality of continuous probability distributions. Given the sex differences in anthropometric measurements and comorbidities, variables were stratified by sex; a Student’s *t*-test or Mann–Whitney *U* test was performed for continuous variables, and an *X*^2^ or Fisher test was performed for categorical variables. Correlation analysis was performed to determine if weight change correlated with an increased executive functioning performance at six months. The multiple regression analysis was used to assess if weight change predicts the performance of EF and global cognition at a 6-months follow-up, adjusted for sex, age, schooling years, and psychopathology; the logarithmic transformation was made for variables that did not have a normal distribution as assessed by Kolmogorov–Smirnov. Moreover, we evaluated the association of baseline cognitive performance with success in weight loss at a 6-month follow-up with an *X*^2^ or Fisher test. Two-sided *p* values were calculated and considered significant when *p* < 0.05. The post hoc multiple comparisons were corrected with the Bonferroni method. SPSS version 22.0 (IBM Inc., Armonk, NY, USA) was used for all analyses.

## 3. Results

### 3.1. Population Characteristics

**Figure 1:**
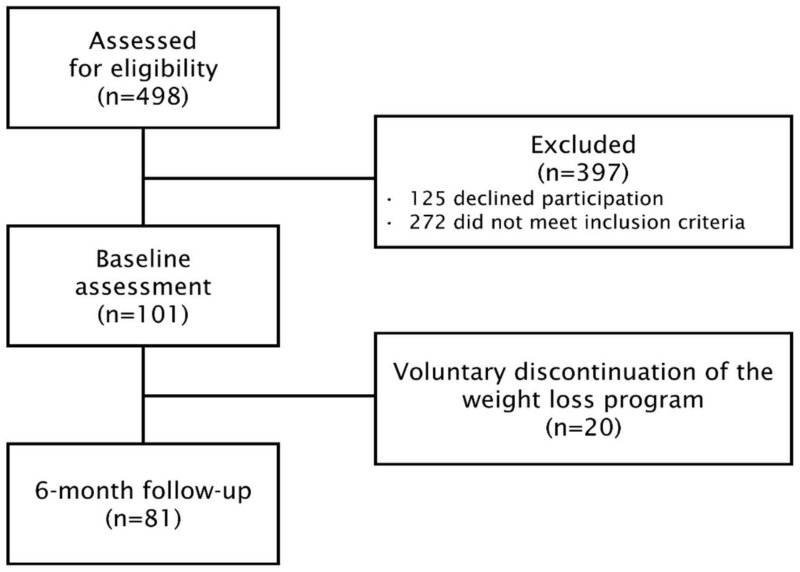
Flow chart of the study cohort.

Among 498 patients screened for potential participation in our longitudinal cognitive assessment program, 101 individuals met the inclusion criteria, and 81 completed the weight loss program and the cognitive evaluations (72% women and 92% men) (Figure 1).

The median age was 40.0 years (IQR: 31.5–47), with 61% women, with a median BMI 41.4 (IQR: 36.7–45.9), pertaining predominantly to the middle class (64%), and 85% had at least high school education or more. The median schooling was 16.0 years (IQR: 12–17), and there was relatively low smoking habit (23%), and moderate alcohol consumption (45.5%), with differences in the frequency of alcohol consumption between men and women (*p* = 0.015). All participants reported low (41%) or moderate (25%) levels of physical activity (Table 1).

The prevalence of metabolic syndrome was 80%, being higher in men compared to women (*p* = 0.045). Hypertension (*p* = 0.0015) and obstructive sleep apnea (*p* = 0.0015) were more frequent in men than in women (Table 1).

As expected, women had a higher percentage of body fat, while men had a higher percentage of fat-free mass and higher waist circumference. At a six-month follow-up, the mean weight loss was 2.67%, with only 29.6% having a successful (i.e., ≥5%) weight loss, although there was a statistically significant decrease in weight (*p* < 0.001), BMI (*p* < 0.001), and waist circumference (*p* < 0.001) in all participants. Nevertheless, women had a higher reduction in fat mass (*p* = 0.008) than men; while men had an increase in fat-free mass (*p* < 0.001), better response on HDL-cholesterol (*p* = 0.043) and systolic blood pressure (*p* = 0.013), as compared to women. Psychopathology prevalence at baseline was similar between women and men. At a six-month follow-up, women had a significant improvement in major depressive disorder (*p* = 0.018), and a higher reduction in anxiety (*p* = 0.031) and depression scores (*p* = 0.021), while men only improved in the depression score (*p* = 0.031) (Table 2).

### 3.2. Cognitive Functioning

At baseline, all patients had a median of MoCA of 26.0 points (IQR: 23.5–28), and the executive functioning score was: dorsolateral PFC 100.0 (IQR: 90–109), anterior PFC 101.0 (IQR: 90–115), medial orbital CPF 97.0 (IQR: 78–106), and global EF 98.0 (IQR: 87–108). At a six-month follow-up, there was an improvement in EF evaluated with BANFE (*p =* 0.0024) and global cognition with MoCA (*p =* 0.0024) (Table 3).

There were no differences in the performance of the MoCA, TMT-B, BANFE-2, or BANFE-NQS batteries between women and men (Table 4). After the multidisciplinary program, the cognitive performance improved, being more pronounced in women, with an increase in dorsolateral PFC, anterior PFC, global PFC, hot EF assessed by BANFE-NQS, and several subdomains, such as verbal working memory, as well as cognitive flexibility. On the other hand, men only improved cognitive flexibility and the total score of the BANFE-2 battery.

**Figure 2:**
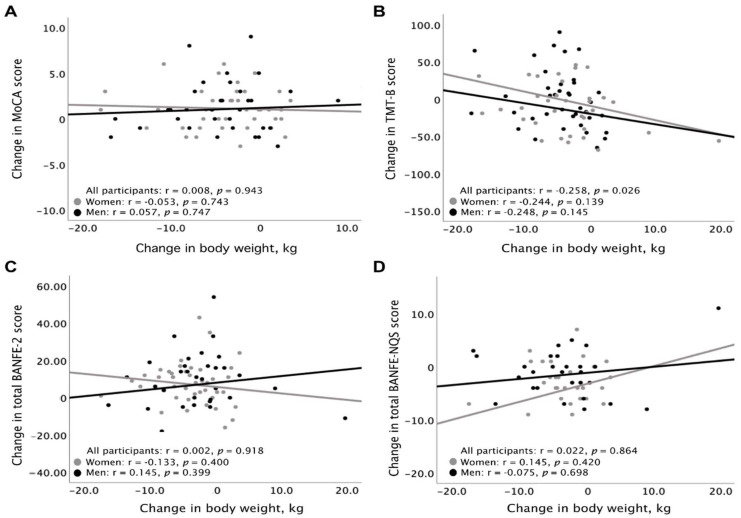
Scatter plot showing the correlation between the change in body weight with change in MoCA score (**A**), change in TMT-B score (**B**), change in BANFE-2 score (**C**), and with change in BANFE-NQS (**D**). MoCA: Montreal Cognitive Assessment; TMT–B: Trail Making Test–Part B; BANFE–2: Neuropsychological Battery of Executive Functions (EF) and Frontal Lobes; BANFE–NQS: Neuropsychological Questionnaire Self–perception.

No significant correlation was found between weight loss and the change in global cognition evaluated, with MoCA and EF evaluated with BANFE-2 and BANFE-NQS. Nonetheless, there was a mild negative correlation (i.e., improvement) between TMT-B and weight change (Figure 2).

In the multiple regression analysis, weight change, sex, age, and psychopathology did not predict cognitive performance; schooling was the only predictor of the total MoCA score at a six-month follow-up (Table 5).

Moreover, there was no association in those individuals with normal or impaired cognitive performance at baseline with successful weight loss in the multidisciplinary weight loss program (Table 6).

## 4. Discussion

In our study, EF and global cognition were found within normal values at baseline; notwithstanding, there was an improvement after a six-month follow-up. Moreover, our patients showed a favorable change in anthropometric parameters, anxiety, and depression scores. The cognitive performance improvement did not correlate with the magnitude of weight loss, except for a negative correlation with TMT-B, which indicates faster information EF processing speed and cognitive flexibility. It is well known that the increase in cognitive flexibility allows the individual the ability to adapt during challenges. This flexibility could benefit patients under obesity treatment to find new strategies and tools, to select and switch to more effective and realistic behavioral and lifestyle changes for weight loss [21].

Although, to our knowledge, there are no observational or interventional studies evaluating the effect of weight loss on cognitive performance in the Mexican population, there are indeed several studies showing that Mexicans with obesity have cognitive performance impairments when compared with lean subjects [36,37]. In other populations, several studies have demonstrated the influence of weight loss programs on cognitive performance, such as diet type [38,39], exercise [40], pharmacotherapy (e.g., metformin) [16], and metabolic control [41]. Napoli et al. studied the cognitive impact in older adults with obesity after one year of randomly assigned weight management, exercise, or weight loss management-plus-exercise; they found each treatment improve cognition, but their combination provided higher benefits [42]. Perhaps the effect of the combination of strategies in a multidisciplinary program such as ours impacts cognition positively. Unfortunately, the attained weight loss in our program was lower than the recommended 5–10% [32], which could also explain the lack of cognitive improvement related directly to weight loss in our program, except for the TMT-B test (*p* = 0.026).

At baseline, there were no differences in cognitive performance between men and women, despite differences in the prevalence of moderate alcohol consumption (50% for men vs. 33% for women). In two different systematic reviews, it was found that light to moderate alcohol use in middle to late adulthood is not associated with risk of cognitive impairment [43,44].

Women experienced a higher improvement in executive functions than men. This might be related to the fact that men had a higher prevalence of metabolic syndrome, hypertension, and sleep apnea, each of which is independently associated with cognitive impairment [12,13,14,45].

The alterations in EF may influence the dietary behavior modulation and the perpetuation of obesity, turning it into a vicious circle [16]. It is also known that some patients with abnormal EF may experience disruptive behavior affecting adherence to medical treatments [46]. Our study did not find an association between normal or impaired baseline cognitive functioning and weight loss success. This finding is in agreement with another study, where patients with mild cognitive impairment can respond correctly to weight loss programs, regardless of baseline cognitive functioning [47].

In the multiple regression analysis, we did not find a significant association between the traditional predictor variables, such as age, sex at birth, BMI, school education, psychopathologies, with weight loss. However, it was found that schooling predicts overall performance in MoCA and TMT-B cognitive tests, both in men and women. Similar findings have also been described in a meta-analysis that evaluated school education and the risk of dementia, finding that the risk of dementia was reduced by 7% for every school year achieved [48].

Our study has several limitations that should be addressed. First, the weight loss goal (>5%) was not attained in most patients, which challenged our hypothesis that the magnitude of weight loss is associated with an improvement in cognitive performance after six months. Second, although the BANFE-2 battery comprises international tests, it is only validated in the Mexican population. Despite the limitations, the present study confirms that obesity is associated with abnormalities in cognitive functioning, even in young adults, and strengthens the hypothesis that weight loss can improve aspects of cognitive performance, especially processing speed and cognitive flexibility. Further interventional studies are necessary to confirm our hypothesis that weight loss improves cognitive functioning in persons with severe obesity.

The study of obesity and its relationship with cognitive impairment and dementia is quite relevant in view of the extended lifespan that the human population is experiencing. Obesity can influence the course not only of Alzheimer’s disease and vascular dementia, but also of less prevalent neurodegenerative forms of dementia such as progressive supranuclear palsy and corticobasal syndrome [15,49,50]. However, whether obesity is an additive factor in cognitive impairment or a direct pathophysiological trigger in neurodegenerative dementias is still a matter of debate.

## 5. Conclusions

In conclusion, patients with obesity showed an improvement in aspects of the cognitive performance after six months in a multidisciplinary structured weight loss program, although the magnitude of weight loss did not correlate with the magnitude of cognitive improvement. This finding could be the consequence of the multidisciplinary intervention as a whole, highlighting the possible benefits of integral programs for obesity treatment.

## Figures and Tables

**Table 1:** Sociodemographic and clinical characteristics.

	All Participants	Women	Men	*p*-Value ^†^
	(*n* = 81)	(*n* = 45)	(*n* = 36)	
Age, median (IQR), years	40.0 (31.5–47)	40 (31.7–46.2)	40.0 (31.0–47.0)	0.99
Education, median (IQR), years	16 (12–17)	15 (12.0–17.0)	16 (12.0–17.0)	0.99
Socioeconomic status, *n* (%)				
Low Middle High	16 (15.8) 64 (63.4) 21 (20.8)	9 (14.5) 40 (64.5) 13 (21.0)	7 (19.9) 24 (61.5) 8 (20.5)	0.99
Education, *n* (%)				
6 years of education 7 to 12 years of education 13 or more years of education	15 (14.9) 30 (29.7) 56 (55.4)	10 (16.1) 20 (32.3) 32 (51.6)	5 (12.8) 10 (25.6) 24 (61.5)	0.99
Cigarette smoking, *n* (%)				
Absent Mild (≤5 cigarettes/day) Moderate (6–15 cigarettes/day)	78 (77.2) 18 (17.8) 5 (6.17)	52 (83.9) 9 (14.5) 1 (1.5)	26 (66.7) 9 (23.1) 4 (11.1)	0.99
Alcohol consumption, *n* (%)				
Absent Mild (W: 1 d/day, M: 2 d/day) Moderate (W: 2 d/day, M: 3 d/day)	55 (54.5) 10 (9.9) 36 (44.4)	41 (66.1) 6 (9.70) 15 (33.3)	14 (35.9) 4 (10.3) 21 (58.3)	**0.015**
Physical activity, *n* (%)				
Mild (<600 Mets/week) Moderate (600–1499 Mets/week) Heavy (≥1500 Mets/week)	42 (41.6) 25 (24.8) 34 (33.7)	26 (41.9) 18 (29.0) 18 (29.0)	16 (41.0) 7(17.9) 16 (41.0)	0.99
Comorbidities, *n* (%)				
Metabolic syndrome	81 (80.2)	44 (71.0)	37 (94.9)	**0.045**
Hypertension	43 (53.1)	22 (35.5)	27 (69.2)	**0.0015**
Prediabetes	44 (43.6)	30 (48.9)	14 (35.9)	0.99
Type 2 diabetes mellitus	25 (30.9)	12 (19.4)	17 (43.6)	0.195
Hypertriglyceridemia	42 (51.9)	27 (44.3)	25 (64.1)	0.99
Hypoalphalipoproteinemia	63 (77.8)	51 (82.3)	29 (74.4)	0.99
Hypercholesterolemia	26 (32.1)	21 (33.9)	15 (38.5)	0.99
Obstructive sleep apnea	65 (80.2)	40 (64.5)	36 (92.3)	**0.0015**

IQR: Interquartile range, W: women, M: men, d: drinks. ^†^ Bonferroni-corrected *p*-values. Statistically significant *p*-values in bold.

**Table 2:** Comparison of anthropometric, biochemical, and psychopathology parameters after six months of a multidisciplinary weight loss program.

	Women (*n* = 45)		Men (*n* = 36)		Sex Comparison
Variables	Baseline	6-Month Follow-Up	Baseline	6-Month Follow-Up	^† ^*p*-Value
**Anthropometric, median (IQR)**					
Weight, kg	108.5 (93.8–116.0)	101.9 (89.5–114.6) ^#^	135.4 (109.8–151.1)	131.6 (104.0–149.0) ^#^	**0.0017**
BMI, kg/m^2^	42.0 (36.4–44.9)	40.1 (34.9–45.5) ^#^	42.3 (36.8–49.5)	40.8 (34.7–49.1) ^#^	0.334
Waist, cm	115.9 (108.1–124.6)	110.0 (103.0–120.0) ^#^	129.5 (115.143.9)	121.7 (115.8–142.8) ^#^	**0.0017**
Fat mass, %	50.5 (47.1–54.0)	49.9 (44.6–53.0) **	42.3 (38.8–46.6)	41.2 (38.2–45.2)	**0.0017**
Fat–free mass, %	49.4 (45.6–52.7)	50.1 (47.0–55.4)	57.8 (53.3–61.3)	58.4 (53.6–61.0) ^#^	**0.0017**
Systolic BP, mmHg	120.0 (110.0–130.0)	120.0 (110.0–120.0)	130.0 (120.0–140.0)	120.0 (120.0–130.0) *	**0.017**
Diastolic BP, mmHg	80.0 (80.0–85.5)	80.0 (70.0–80.0) **	89.0 (80.0–95.0)	80.0 (80.0–85.7) **	0.068
**Biochemical, median (IQR)**					
Glucose, mg/dL	91.0 (83.5–103.5)	91.0 (85.0–99.7)	100.5 (83.5–114.7)	95.0 (82.0–110.0)	0.99
Total cholesterol, mg/dL	143.0 (111.0–206.5)	159.0 (107.5–198.2)	150.5 (130.7–267.2)	178.0 (136.0–230.0)	0.99
LDL cholesterol, mg/dL	108.0 (97.0–129.2)	104.0 (91.5–112.0)	111.5 (87.2–135.0)	108.0 (95.0–136.0)	0.99
HDL cholesterol, mg/dL	44.0 (37.5–49.0)	44.0 (39.2–50.7)	34.0 (32.0–40.7)	36.5 (31.7–43.0) *	**0.0017**
Triglycerides, mg/dL	108.0 (97.0–129.2)	104.0 (91.5–112.0)	111.5 (87.2–135.0)	108.0 (95.0–136.0)	0.99
**Psychopathology, *n* (%)**					
Depressive disorder	9 (20.0)	3 (6.70) *	3 (8.30)	5 (13.9)	0.595
Anxiety disorder	17 (37.8)	11 (24.4)	14 (38.9)	12 (33.3)	1.0
Binge eating	9 (20.0)	5 (11.1)	11 (30.6)	5 (13.9)	0.99
**HAD scale, median (IQR)**					
Anxiety, score	8.0 (3.0–10.7)	5.0 (3.0–7.0) *	7.0 (5.0–10.2)	6.0 (3.0–7.75) *	0.99
Depression, score	5.50 (3.0–90)	4.0 (3.0–6.7) *	7.0 (4.2–8.0)	6.0 (4.0–8.0)	0.99

BMI: Body Mass Index; BP: Blood pressure; HAD: Hospital Anxiety and Depression Scale; IQR: Interquartile Range. Wilcoxon rank and McNemar test were realized to find the differences at baseline and a six-months follow-up, * *p* < 0.05, ** *p* < 0.01, ^#^ *p* < 0.0001. ^†^ A Mann–Whitney *U*, X^2^, or Fisher test was performed to test the differences according to sex at baseline. ^†^ Bonferroni-corrected *p*-values. Statistically significant *p*-values in bold.

**Table 3:** Comparison of cognitive performance at baseline and after six months in a multidisciplinary weight loss program.

		All Participants (*n* = 81)		^† ^*p*-Value
Reference Value		Baseline	6-Month Follow-Up	
MoCA, median (IQR), score	26–30	26.0 (23.5–28.0)	27.0 (25.0–28.0)	**0.0024**
TMT-B	75–273	85.0 (63.0–106.5)	73.0 (58.0–100.0)	0.99
BANFE-2, median (IQR), score				
Medial orbital PFC	80–115	97.0 (78.5–106.5)	100.0 (85.0–112.0)	0.888
Dorsolateral PFC	80–115	100 (90.0–109.5)	103.0 (94.0–113.0)	**0.0024**
Anterior PFC	80–115	101 (90–115.5)	106.0 (97.0–118.0)	0.168
Total Score	80–115	98.0 (87.0–108.0)	103.0 (92.0–116.0)	**0.0024**
BANFE-2 performance grading, *n* (%)				
Severe	≤69	6 (7.70)	5(6.40)	1.00
Mild to moderate	70–84	12 (14.8)	13 (16.7)	0.99
Normal	85–115	52 (64.2)	54 (66.7)	0.99
Superior	≥116	11 (12.8)	9 (10.3)	0.99
BANFE-NQS, median (IQR), score				
Self-consciousness	<3	1.0 (0.0–1.0)	0.0 (0.0–1.0)	**0.048**
Interests and motivations	<4	1.0 (0.0–3.0)	0.0 (0.0–1.7)	**0.0024**
Behavioral Control	<4	3.0 (1.0–4.5)	2.0 (1.0–3.0)	0.096
Frustration Tolerance	<3	1.0 (0.0–2.0)	1.0 (0.0–1.0)	0.552
Mood	<3	1.0 (0.0–2.0)	0.0 (0.0–1.0)	0.696
Executive Functioning	<4	2.0 (1.0–3.2)	2.0 (1.0–3.0)	0.99
Total score	<15	10.0 (5.5–14.5)	6.0 (3.0–10.0)	**0.0024**
EF Subdomain, median (IQR), score				
Planning		11.0 (9.3–12.0)	11.0 (9.91–12.0)	0.99
Decision Making		10.0 (8.0–12.0)	10.2 (8.37–13.0)	0.99
Verbal WM		8.66 (7.6–10.0)	9.16 (8.29–10.5)	0.096
Visuospatial WM		9.83 (9.0–11.0)	9.83 (9.0–10.7)	0.99
Cognitive Flexibility		12.0 (9.66–13.0)	12.5 (11.3–14.0)	**0.024**
Inhibitory Control		9.87 (8.65–10.7)	10.3 (9.50–11.0)	0.99
Metacognition		10.0 (8.5–11.5)	10.5 (9.50–11.0)	0.99

IQR: Interquartile Range, MoCA Montreal Cognitive Assessment, TMT-B Trail Making Test-Part B, BANFE-2 Neuropsychological Battery of Executive Functions (EF) and Frontal Lobes, PFC Pre-frontal Cortex, BANFE-NQS Neuropsychological Questionnaire Self-perception, WM working memory. ^†^ Bonferroni-corrected *p*-values. Statistically significant *p*-values in bold.

**Table 4:** Comparison of cognitive performance after six months of a multidisciplinary weight loss program by sex.

	Women		Men		Sex Comparison
	Baseline	6 Months	Baseline	6 Months	^† ^*p*-Value
MoCA, median (IQR)	26.0 (24.0–28.0)	27.0 (25.0–28.0) *	26.5 (23.0–28.0)	27.0 (24.7–28.0) *	0.99
TMT–B, median (IQR)	83.0 (56.7–94.5)	74.0 (60.0–99)	91.5 (68.0–125.7)	71.0 (55.0–101.5) *	0.99
BANFE–2, median (IQR)					
Medial orbital PFC	97.0 (80.5–105.2)	103.0 (83.0–112.0)	100.0 (765–107.0)	100.0 (85.2–112.0)	0.99
Dorsolateral PFC	99.0 (81.5–110.0)	103.0 (90.0–111.0) ^&^	102.0 (91.2–109.7)	103.5 (96.0–113.0)	0.99
Anterior PFC	97.0 (85.5–111.0)	104.0 (95.0–118.0) *	105.0 (95.5–118.0)	108.5 (104.0–118.0)	0.99
Total score	96.0 (83.5–111.0)	104.0 (90.0–113.0) ^&^	101.5 (87.5–107.0)	102.0 (93.5–117.7) *	0.99
BANFE–2 global performance, *n* (%)					
Severe	5 (11.9)	2 (4.80)	1 (2.80)	3 (8.30)	0.99
Mild to moderate	7 (15.4)	10 (22.0)	4 (12.1)	4 (12.1)	1.0
Normal	26 (57.1)	30 (65.5)	27 (73.0)	24 (65.7)	0.99
Superior	7 (15.6)	3 (7.80)	4 (12.1)	5 (13.9)	1.0
BANFE–NQS, median (IQR)					
Self–consciousness	1.0 (0.0–1.0)	0.0 (0.0–1.0) **	1.0 (0.0–1.0)	0.0 (0.0–0.5)	0.99
Interests & motivations	1.0 (0.0–3.0)	0.0 (0.0–1.0) ^&^	1.0 (0.0–3.0)	0.0 (0.0–2.0)	0.99
Behavioral Control	3.0 (1.0–5.0)	1.0 (0.5–3.0) ^#^	1.0 (1.0–4.0)	2.0 (1.0–3.0)	0.88
Frustration Tolerance	2.0 (0.0–3.0)	1.0 (0.0–1.0) **	1.0 (0.0–2.0)	1.0 (0.0–1.0)	0.378
Mood	1.0 (0.0–3.0)	0.0 (0.0–2.0) *	1.0 (0.0–1.0)	0.0 (0.0–1.0)	0.192
Executive Functioning	2.0 (1.0–4.0)	2.0 (1.0–3.5)	1.0 (1.0–3.0)	1.0 (1.0–2.5)	0.888
Total score	11.5 (6.7–16.2)	5.0 (3.0–9.5) ^#^	7.0 (2.0–13.0)	6.0 (3.0–10.0)	0.576
EF Subdomain, median (IQR)					
Planning	10.6 (9.33–12.0)	10.6 (8.33–12.0)	11.3 (9.0–12.0)	11.3 (10.33–12.0)	0.99
Decision Making	9.50 (7.50–11.2)	9.50 (7.50–12.5)	10.5 (8.0–12.0)	11.0 (8.75–13.2)	0.99
Verbal WM	8.75 (7.79–9.91)	9.16 (8.16–10.7) **	8.5 (7.62–10.3)	9.33 (8.25–10.5)	0.99
Visuospatial WM	10.3 (9.00–11.1)	10.3 (9.08–10.6)	9.7 (8.87–10.8)	9.66 (9.00–11.0)	0.99
Cognitive Flexibility	12.0 (8.16–13.3)	12.6 (11.0–14.0) *	11.8 (9.75–12.6)	12.3 (11.5–13.8) **	0.99
Inhibitory Control	9.87 (8.87–10.8)	10.3 (9.25–11.2)	9.87 (8.37–10.7)	10.5 (8.37–11.2)	0.99
Metacognition	9.50 (8.0–11.0)	10.0 (8.50–11.0)	10.2 (9.50–11.5)	10.5 (9.50–11.5)	0.192

IQR: Interquartile Range, MoCA: Montreal Cognitive Assessment; TMT–B: Trail Making Test–Part B; BANFE–2: Neuropsychological Battery of Executive Functions (EF) and Frontal Lobes; PFC: Pre–frontal Cortex; BANFE–NQS: Neuropsychological Questionnaire Self–perception; WM: working memory. Wilcoxon rank and McNemar test were realized to find the differences at baseline and follow up, * *p* < 0.05, ** *p* < 0.01, ^&^ *p* < 0.001, ^#^ *p* < 0.0001. ^†^ A Mann–Whitney *U*, X^2^, or Fisher test was performed to test the differences by sex at baseline. ^†^ Bonferroni-corrected *p*-values.

**Table 5:** Prediction of global cognitive performance and executive functioning at the end of a multidisciplinary weight loss program.

	B	Exp (B)	*p*-Value
MoCA (score < 26) ^a^			
Constant	2.624	13.790	0.151
Female sex	−0.127	0.881	0.812
Age	−0.013	0.987	0.667
Weight change	−0.041	0.960	0.409
Education years	−0.207	0.813	0.007
Psychopathology	−0.072	0.931	0.894
BANFE-2 (score < 85) ^b^			
Constant	0.679	1.973	0.785
Female sex	−0.091	0.913	0.903
Age	−0.005	0.995	0.893
Weight change	−0.051	0.950	0.397
Education years	−0.167	0.847	0.110
Psychopathology	−0.910	0.402	0.245
BANFE-NQS (score < 15) ^c^			
Constant	−1.095	0.335	0.736
Female sex	−0.857	0.424	0.377
Age	−0.036	0.964	0.459
Weight change	0.032	1.033	0.635
Education years	0.122	1.020	0.874
Psychopathology	1.636	5.133	0.151

MoCA Montreal Cognitive Assessment, BANFE-2 Neuropsychological Battery of Executive Functions and Frontal Lobes, BANFE-NQS Neuropsychological Questionnaire Self-perception, Exp (B) odds ratio, B regression coefficient beta. Multiple regression analysis. **^a^** Cox & Snell R^2^ = 0.112, Nagelkerke R^2^ = 0.156, *p* = 0.007. ^b^ Cox & Snell R^2^ = 0.055, Nagelkerke R^2^ = 0.108, *p* = 0.485. ^c^ Cox & Snell R^2^ = 0.060, Nagelkerke R^2^ = 0.136, *p* = 0.712.

**Table 6:** Association of baseline cognitive performance with success in weight loss at a six-month follow-up.

		Weight Loss ≥ 5%	Weight Loss < 5%	Weigh Gain ≥ 0.1%	*p*-Value
Neurocognitive Test	ReferenceScore	(*n* = 24)	(*n* = 38)	(*n* = 19)	
MoCA performance					
- MoCA normal	≥26	17 (69.6)	18 (48.6)	11 (58.8)	
- MoCA impaired	<26	7 (30.4)	20 (51.4)	8 (41.2)	0.272
TMT-B performance					
- TMT-B normal	≤273	21 (100)	35 (100)	19 (100)	
- TMT-B impaired	>273	0 (0.0)	0 (0.0)	0 (0.0)	-
BANFE-2 performance					
- BANFE-2 global normal	≥85	19 (79.2)	30 (78.9)	16 (84.2)	
- BANFE-2 global impaired	<85	5 (20.8)	8 (21.1)	3 (15.8)	0.661
- Medial orbital PFC normal	≥85	20 (83.3)	25 (68.8)	12 (63.2)	
- Medial orbital PFC impaired	<85	4 (16.7)	13 (31.2)	7 (36.8)	0.565
- Dorsolateral PFC normal	≥85	17 (70.8)	30 (78.9)	17 (89.5)	
- Dorsolateral PFC impaired	<85	7 (29.2)	8 (21.1)	2 (10.5)	0.356
- Anterior PFC normal	≥85	21 (87.5)	27 (77.1)	18 (94.7)	
- Anterior PFC impaired	<85	3 (12.5)	11 (22.9)	1 (5.30)	0.216
BANFE–NQS performance					
- BANFE–NQS normal	<15	19 (79.2)	29 (76.3)	16 (84.2)	
- BANFE–NQS impaired	≥15	5 (20.8)	9 (23.7)	3 (15.8)	0.870

MoCA: Montreal Cognitive Assessment; TMT–B: Trail Making Test–Part B; BANFE–2: Neuropsychological Battery of Executive Functions (EF) and Frontal Lobes; PFC: Pre–frontal Cortex; BANFE–NQS: Neuropsychological Questionnaire Self–perception.

## Data Availability

The data sets generated for the present study will be available from the corresponding author upon request.

## Supplementary Materials

Supplementary materials are available at https://www.mdpi.com/article/10.3390/brainsci12040509/s1, Table S1: Conceptual map of *BANFE-2* Neuropsychological Battery of Executive Functions and Frontal Lobes; Table S2: Conceptual map of subdomain executive functions with some of the tasks of BANFE-2 battery.
