# Supplementary material for: Influence of Weight Loss on Cognitive Functions: A Pilot Study of a Multidisciplinary Intervention Program for Obesity Treatment
Source: Brain Sci. 2022 Apr 17;12(4):509. doi: 10.3390/brainsci12040509 (PMC9028728; doi:10.3390/brainsci12040509)
Supplement: Supplementary file 1 [file brainsci-12-00509-s001.zip › brainsci-1624651-supplementary.pdf]

Supplementary Materials:

Supplementary Table S1. Conceptual map of *BANFE-2* Neuropsychological Battery of Executive Functions and Frontal Lobes

**Anterior PFC**	**Features evaluated**<br>- Metamemory<br>- Figurative meaning comprehension<br>- Abstraction abilities<br><br>**Description of the tests that are included in BANFE-2**<br>1. *Semantic classification task*. Assesses productivity, or the ability to categorize objects into different semantic groups according to their similarities. This task also assesses understanding abstract information and creating categories based on abstract concepts.<br>2. *Proverb comprehension*. Assesses the ability to understand, compare and select sentences with figurative sense.<br>3. *Metamemory*. Assesses the ability to develop a learning and memory strategy (metacognitive control), the ability to make predictions about one's own memory performance (metacognitive judgement), and to compare the predicted performance with the actual memory capacity (metacognitive self-monitoring).
**Dorsolateral PFC**	**Features evaluated**<br>- Verbal fluency<br>- Productivity<br>- Visuospatial planning<br>- Sequential planning<br>- Cognitive flexibility<br>- Inverse sequentiation<br>- Working memory (verbal and visuospatial)<br>- Control of encoding processes<br>**Description of the tests that are included in BANFE-2**<br>1. *Self-ordered pointing task.* Assesses the ability to plan a solving strategy and keep in mind and update visuospatial information. The individual is asked to point at -without repetition- a set of images and to keep record of those that were already pointed at.<br>2. *Sequential visuospatial working memory task*. This task is analogous to the Corsi task, but drawings of common objects displayed on a sheet of paper are used instead of cubes. The task offers a measure of visuospatial working memory by assessing the ability to remember and reproduce the order in which a set of drawings on the sheet of paper are pointed at.<br>3. *Verbal working memory – word ordering task.* Assesses the ability to maintain and work with verbal information in the mind.<br>4. *Card sorting task*. Assesses the ability to think flexibly and to create classification hypotheses and problem-solving strategies.<br>5. *Maze-solving task*. Assesses the ability to anticipate, plan and solve visuospatial problems.<br>6. *Tower of Hanoi.* Assesses the ability to plan and organize responses in sequential order to solve a visuospatial and manipulative problem.<br>7. *Consecutive subtraction*. This is a working memory task that assesses the ability to mentally maintain, update and work with numerical information.

	8. *Verbal fluency task (verbs).* This task requires executive and verbal functions. It measures the ability to produce as many words as possible from a particular category (verbs) in a given time.
**Orbitofrontal and Medial orbital PFC**	**Features evaluated**<br>- Inhibitory control<br>- Rule abiding<br>- Risk-benefit assessment<br>- Decision-making<br><br>**Description of the tests that are integrated in BANFE-2 battery**<br>1. *Stroop*. This test measures inhibitory control and attention.<br>2. *Card game task*. This task is based on the Iowa Gambling Task, and allows to assess the subject's ability to make advantageous decisions, and to detect and avoid risky choices.<br>3. *Maze-solving task*. This task also measures inhibitory control and the ability to follow instructions and rules.

PFC Prefrontal cortex. Flores-Lazaro J, et al. (2008) Batería de funciones frontales y ejecutivas: Presentación. Rev Neuropsicología, Neuropsiquiatría y Neurociencias 8(1):141-58 [35].

Supplementary Table S2. Conceptual map of subdomain executive functions with some of the tasks of BANFE-2 battery

**Subdomain**	**Task**
Planning	*Maze-solving task*<br>*Tower of Hanoi*: 3-disk tower total movements and, 4-disk tower total movements<br>Planning errors: number of times the subject reached a non-escape end<br>Time of completion / speed
Decision Making	*Card game task* (based on the Iowa Gambling Task): percentage of disadvantageous choices and, card game task net score.
Verbal WM	*Alphabetical ordering*: the subject must recall in alphabetical order a list of words. There are three different lists of 5 trials each. Scores are obtained based on the minimum number of attempts required to successfully recall all words in the correct order, perseverative errors, order errors, and intrusive words (words that were not on the list).<br>*Substractions*: 40-3 and 100-7<br>Hits: correct individual consecutive substraction.<br>*Backward digit span task*
Visuospatial WM	*Self-ordered pointing task*<br>-Hits: total number of items pointed by the participant following the rules of the task (For this task, scores were obtained according to the number of hits, perseverative errors, and omissions of items).<br>-Perseverative errors: items that the participant previously pointed<br>-Omissions: non-pointed items.<br>*Visuospatial working memory task*: based on the Corsi Cubes task. Instead of cubes, the subject must point in the correct order, as shown by the evaluator, a set of images displayed on a sheet (For this task, scores were obtained according to the highest number of items correctly recalled, perseverations and errors of order).
Cognitive Flexibility	*Card sorting task*:<br>-Hits: total number of cards sorted in the correct category.<br>-Perseverative errors: failure to switch to another category.<br>-Deferred perseverative errors: when the subject chooses the same wrong category that was previously picked in any of the preceding four attempts.
Inhibitory Control	*Stroop colour and word task*. BANFE-2 includes two versions, adapted from the Stroop task:<br>-Version A: the subject must read the words as fast as possible and say the name of the colour of the ink when the words are outlined.<br>-Version B: the subject must switch between reading the word in the first column and then naming the colour of the ink in the next column, and so on (For both Stroop versions, scores are obtained based on the total number of hits, Stroop errors and non-Stroop errors).<br>*Maze solving*<br>Line-crossing: times the subject crossed any line of the maze (against the rules)
Metacognition	*Metamemory task*<br>Negative errors: when the subject's performance prediction underestimates the total number of words they can remember.<br>Positive errors: when the subject's performance prediction overestimates the total number of words they can remember.

WM working memory, *TMT-B* Trail Making Test-Part B, *BANFE-2* Neuropsychological Battery of Executive Functions and Frontal Lobes. There are no cut-off scores of normality for these tasks; nevertheless, a higher score reflects a better cognitive performance
